# Supplementary material for: Antibody Recognition of CD4-Induced Open HIV-1 Env Trimers
Source: J Virol. 2022 Nov 30;96(24):e01082-22. doi: 10.1128/jvi.01082-22 (PMC9769388; doi:10.1128/jvi.01082-22)
Supplement: Supplemental file 1 — Fig. S1 and S2 and Tables S1 to S3. Download jvi.01082-22-s0001.pdf, PDF file, 0.5 MB [file jvi.01082-22-s0001.pdf]

Supplemental Material

Antibody recognition of CD4-induced open HIV-1 Env trimers

Zhi Yang[a], Kim-Marie A. Dam[a], Jonathan M. Gershoni[b], Susan Zolla-Pazner[c], Pamela J. Bjorkman[a#]

[a]Division of Biology and Biological Engineering, California Institute of Technology, Pasadena, CA 91125, USA
[b]Shmunis School of Biomedicine and Cancer Research, Tel Aviv University, Israel
[c]Department of Medicine, Division of Infectious Diseases, Icahn School of Medicine at Mount Sinai, New York, NY, 10029 USA

Zhi Yang and Kim-Marie A. Dam contributed equally to this work. Author order was determined by the two co-first authors.

[#]Address correspondence to Pamela J. Bjorkman, bjorkman@caltech.edu

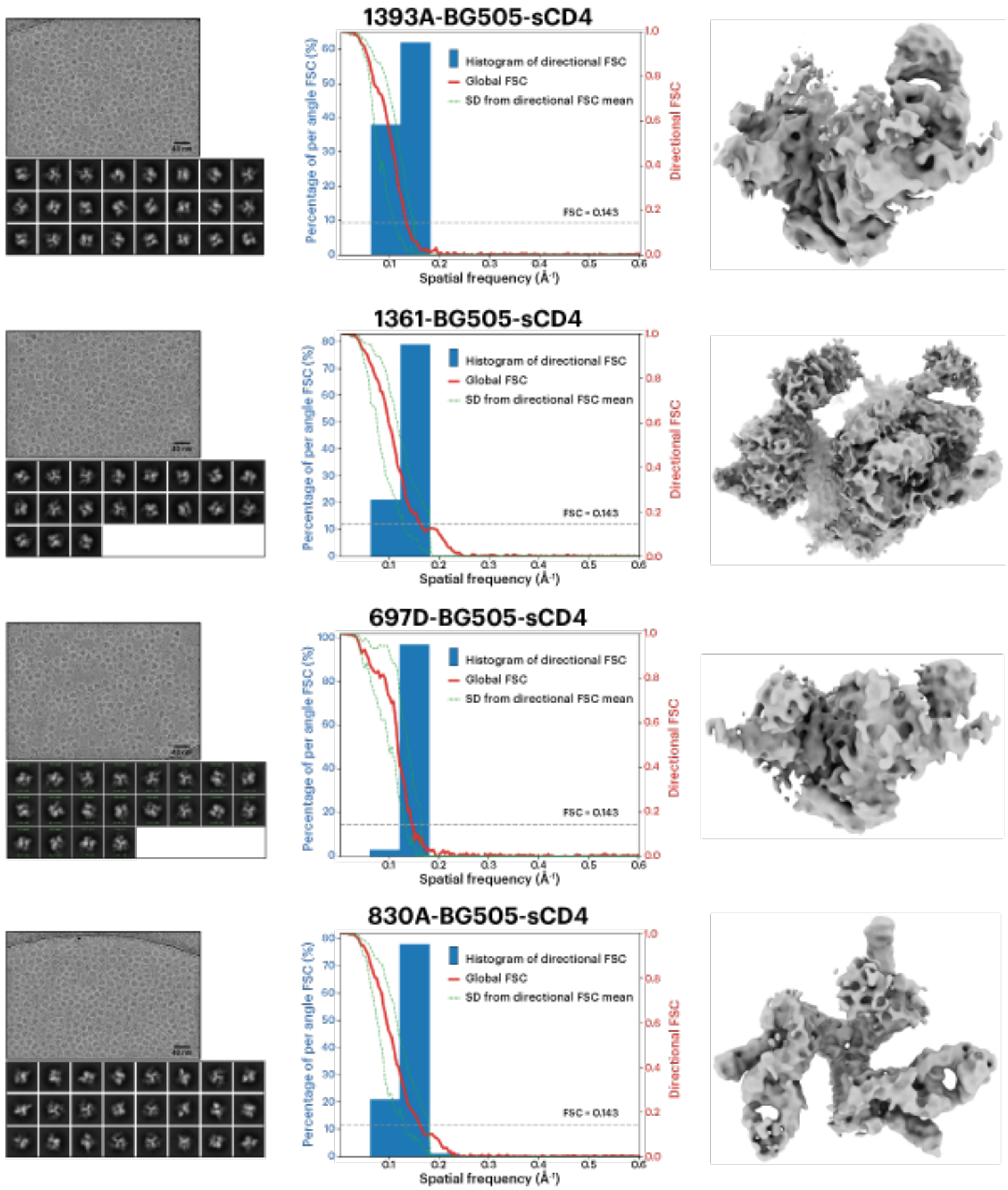

Figure S1. Cryo-EM data processing and validation of V2i Fab-BG505-sCD4 complexes. Left: Example micrographs and 2D class averages of V2i Fab-BG505-sCD4 complexes. Middle: Plots of global half-map FSCs (solid red line), directional resolution values ±1σ from the mean

(left axis, green dashed lines) and distributions sampled over the 3D FSC (blue histograms, right axis). Right: Cryo-EM density maps of V2i Fab-BG505-sCD4 complexes.

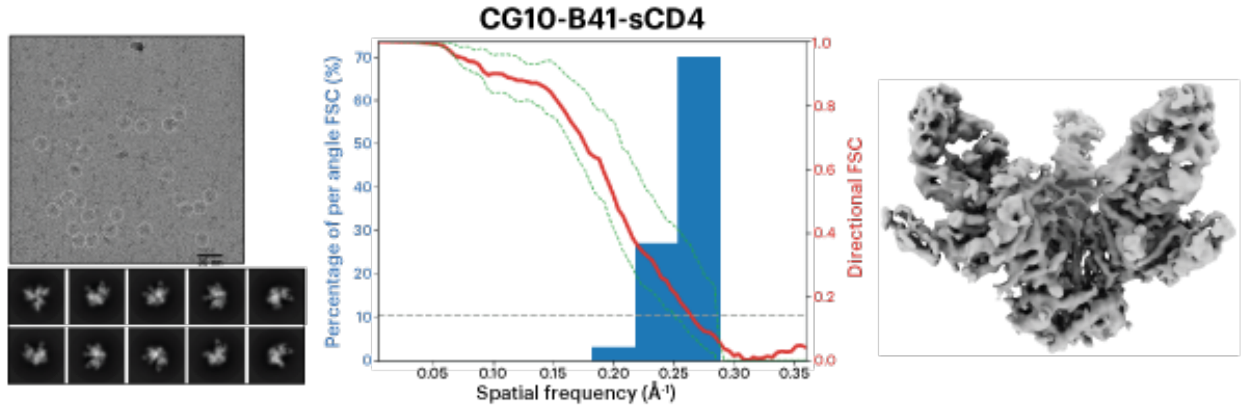

Figure S2. Cryo-EM data processing and validation of CG10 Fab-B41-sCD4 complex. Left: Example micrograph and 2D class averages of the CG10-B41-sCD4 complex. Middle: Plot of global half-map FSC (solid red line), directional resolution values ±1σ from the mean (left axis, green dashed lines) and distributions sampled over the 3D FSC (blue histograms, right axis). Right: Cryo-EM map of the CG10-B41-sCD4 complex.

Supplementary Table 1. Cryo-EM data collection, refinement, and validation statistics

	1393A-BG505-sCD4 (EMD-27209)	1361-BG505-sCD4 (EMD-27210)	697D-BG505-sCD4 (EMD-27211)	830A-BG505-sCD4 (EMD-27212)
**Data collection and processing**				
Magnification *	105,000x	105,000x	105,000x	105,000x
Voltage (kV)	300	300	300	300
Electron exposure (e–/Å^2)	60	60	60	60
Defocus range (μm)	1.8-3.0	1.8-3.0	1.8-3.0	1.8-3.0
Pixel size (Å)	0.416	0.416	0.435	0.416
Recording mode	Super resolution	Super resolution	Super resolution	Super resolution
Collected movies (no.)	3,239	2,412	2,340	1,872
Symmetry imposed	C1	C1	C1	C1
Initial particle images (no.)	943,748	843,403	367,952	436,527
Final particle images (no.)	107,234	104,002	21,655	16,575
Overall map resolution (Å)	7.5	6.1	7.0	7.3

* Nominal magnification

Supplementary Table 2. X-ray data collection and refinement statistics

	CG10 Fab (PDB 8D54)
**Data collection**	
Space group	$P2_12_12_1$
Cell dimensions	
a, b, c (Å)	75.14, 77.80, 84.03
α, β, γ (°)	90, 90, 90
Resolution (Å)	38.90 - 1.40 (1.42 - 1.40) *
R_{merge} (%)	6.5 (151.3)
R_{pim} (%)	1.8 (43.3)
$CC_{1/2}$ (%)	99.9 (63.2)
$I / \sigma I$	19.6 (1.7)
Completeness (%)	99.5 (97.1)
Multiplicity	13.3 (12.5)
**Refinement**	
Resolution (Å)	1.40
No. reflections	97,069 (4,660)
R_{free} / R_{work} (%)	18.8 / 16.9
No. atoms	
Protein	3,330
Ligand/ion	0
Water	680
R.m.s. deviations	
Bond lengths (Å)	0.008
Bond angles (°)	0.86
Rotamer outliers (%)	0.81
Ramachandran plot	
Favored (%)	98.2
Allowed (%)	1.8
Disallowed	0
Average *B*-factor ($Å^2$)	18.9

*Values in parentheses are for highest-resolution shell.

Supplementary Table 3. Cryo-EM data collection, refinement, and validation statistics

	**CG10-B41-sCD4 (PDB 8D5C) (EMD-27208)**
**Data collection and processing**	
Magnification *	105,000x
Voltage (kV)	300
Electron exposure (e–/Å^2)	60
Defocus range (μm)	1.8-3.0
Pixel size (Å)	1.11
Recording mode	Counting
Collected movies (no.)	3,528
Symmetry imposed	C1
Initial particle images (no.)	933,854
Final particle images (no.)	134,746
Overall map resolution (Å) ** (masked/unmasked)	4.1 (4.2)
**Refinement**	
Initial models used (PDB code)	8D54 (this study), 5VN3 ***
Map and model CC	0.66
Map sharpening *B* factor (Å^2)	109.7
Model composition	
Protein residues	2,730
Validation	
MolProbity score	2.43
Clashscore	31.6
Poor rotamers (%)	0
Ramachandran plot	
Favored (%)	93.0
Allowed (%)	7.0
Disallowed (%)	0
RMS deviations	
Length (Å)	0.002
Angles (°)	0.48

* Nominal magnification; ** FSC threshold 0.143; *** Partial structure composed of trimeric gp120/gp41 and sCD4
